# 5-year long-term efficacy of 120-W GreenLight photoselective vaporization of the prostate for benign prostate hyperplasia

**DOI:** 10.1371/journal.pone.0184442

**Published:** 2017-09-13

**Authors:** Juhyun Park, Sung Yong Cho, Min Chul Cho, Hyeon Jeong, Hwancheol Son

**Affiliations:** Department of Urology, Seoul National University College of Medicine, SMG-SNU Boramae Medical Center, Seoul, Korea; Carolina Urologic Research Center, UNITED STATES

## Abstract

**Purpose:**

To investigate 5-year long-term postoperative efficacy in benign prostate hyperplasia (BPH) following 120-W GreenLight high-performance system photoselective vaporization of the prostate (HPS-PVP)

**Methods:**

This was a retrospective study of surgical outcomes in 159 men who underwent HPS-PVP and were followed over 60 months postoperatively. Definitions of treatment success were established based on the following three variables: international prostate symptom scores (IPSS), maximum flow rates (Q_max)_, and quality of life scores QoL). Logistic regression analyses were performed to determine predictors of the postoperative success.

**Results:**

Postoperative IPSS/QoL, Q_max_ and post-voided residual urine volume were significantly improved after HPS-PVP. Postoperative Prostate specific antigen and prostate volume were also well reduced and sustained for 5 years. The postoperative success rate was assessed as 82.1%, 80.8% and 76.1% for each 1-, 3-, and 5-year. Thirty-eight (23.9%) patients had immediate postoperative complications, which were managed successfully with nonsurgical methods. None required transfusions, two (1.2%) patients required endoscopic reoperation for postoperative voiding difficulty due to bladder neck contracture or urethral stricture, and five (3.1%) required HPS-PVP reoperation. Presence of diabetes, voiding symptom subscore, QoL, maximal cystometric capacity, and bladder outlet obstructive index were valuable preoperative parameters for predicting postoperative success.

**Conclusions:**

HPS-PVP is an effective, long-term treatment option for BPH, with sustained efficacy of 76.1% at 5-year follow up. Several preoperative parameters could help to predict the durable surgical improvements.

## Introduction

Benign prostatic hyperplasia (BPH) has been the main cause of low urinary tract symptoms in aging men and it was often associated with significant morbidity [1]. Recently, it has been known combination medical treatments using several type of drugs could achieve effective symptom improvements of BPH [2]. Nevertheless, surgical treatments should be selected primarily in the cases with refractory urinary retention, renal insufficiency, recurrent urinary tract infections, bladder stones or consistent prostate bleeding. In addition, when the medical treatments was not effective, surgery of BPH could be considered and weighed [3].

Transurethral prostatectomy (TURP) has long been accepted as the gold standard for the surgical treatment of BPH, and many clinicians have reported excellent short-term and long-term outcomes [4, 5]. However, TURP is associated with potential risks, including postoperative bleeding requiring transfusions, prolonged urethral Foley catheter indwelling, and transurethral syndrome [6]. To overcome these risks, alternative surgical procedures, including laser techniques, have been developed [7–10]. Laser technologies were first deployed in the early 1990s, Early-generation lasers could relieve bladder outlet obstruction due to BPH by utilizing coagulative and ablative modalities. Ablative techniques, such as GreenLight photoselective vaporization of the prostate (PVP), have remained popular.

The aim of GreenLight PVP is to make an unobstructed channel within the prostate, similar to TURP [11]. Because PVP was basically mimicking TURP, it had the advantage that urologists who had ever experienced TUR surgery could easily learn and practice PVP technique. The learning curves for GreenLight PVP were almost unnecessary [12]. Furthermore, by applying the wedge resection and enucleation technique, adventurous attempts to use GreenLight PVP even in the large size prostate [13, 14].

In recent years, Greenlight system with higher-energy lasers has been available, several expert groups published comparable or even better short-term and medium-term follow-up results using the GreenLight system than other BPH surgical modalities [15, 16]. However, long-term results obtained with the 120-W GreenLight high-performance system (HPS) have rarely been reported yet. Therefore, the aim of the present study was to investigate 5-year long-term surgical efficacy in BPH following 120-W GreenLight HPS-PVP and predictors of postoperative success.

## Methods

The study consisted of 159 patients who underwent HPS-PVP for symptomatic BPH from January 2008 to March 2010 and were followed up for more than 5 years.

The indications for surgery were an International Prostate Symptom Score (IPSS) of 12 points or more before surgery and a maximal urinary flow rate (Q_max_) lower than 15 ml/s, acute urinary tract obstruction, hydronephrosis or uremia, recurrent urinary tract infections due to excessive residual urine, bladder stones, and severe hematuria. The exclusion criteria were a neurogenic bladder in urodynamic studies, urethral stricture, previous prostate surgery, and prostate malignancy. Patients with incomplete preoperative and postoperative follow-up clinical information were also excluded. The study protocol was approved by the Institutional Review Board at Boramae Hospital, and the study conformed to the tenets of the Declaration of Helsinki.

All the patients underwent preoperative evaluations, including transrectal ultrasonography and multichannel video urodynamics (MMS UD-2000, Medical Measurement System, Enscheded, the Netherlands). Medical histories were taken to detect the presence of lower urinary tract symptoms suggestive of BPH, and all the patients underwent physical examinations. Data on age, serum prostate specific antigen (PSA), IPSS, quality of life (QoL), Q_max_, postvoided residual urine volume (PVR), and urodynamic parameters, including the bladder outlet obstruction (BOO) index and bladder compliance, were recorded.[17, 18] In cases of prostate biopsies, these were performed preoperatively or intraoperatively via transrectal ultrasonography guidance. The same surgeon (H.S.) performed all the operations.

The HPS-PVP used in the study has been described in a previous study.[15] Perioperatively, the operative time, total amount of energy used (kJ), Foley catheter maintenance period, immediate complications, and the management of these were recorded. Postoperative complications were reported at every follow-up visit and classified according to the Clavien–Dindo system.

The following were assessed at the preoperative visit and at follow-visits 6, 12, 24, 36, 48, and 60 months after the surgery: IPSS/QoL, Q_max_/PVR, prostate volume, and PSA. The presence of postoperative complications was verified at every follow-up visit.

In this study, definitions of treatment success were established based on the following three variables: IPSS, Q_max_, and QoL.[19, 20] Overall efficacy was defined as the median efficacy grades from the symptoms, function, and QoL domains. Treatment success was defined if the overall efficacy demonstrated an improvement that was “fair or greater”. Univariate and multivariate logistic regression analyses were conducted to determine the predictors of a postoperative success at each 1-, 3-, and 5-year follow up.

## Ethical standards

This study design and the use of patients’ information stored in the hospital database were approved by the institutional Review Board (IRB) at the Seoul Metropolitan Government—Seoul National University Boramae Medical Center. The approval number is 26-2016-123. We were given exemption from getting informed consents by the IRB because the present study is a retrospective study and personal identifiers were completely removed and the data were analyzed anonymously. Our study was conducted according to the ethical principles laid down in the 1964 Declaration of Helsinki and its later amendments.

### Statistical analysis

Variables were presented as mean ± standard deviation. Comparing the pre- and postoperative clinical variables, an analysis of variance or a paired *t*-test and Wilcoxon’s signed-rank test were used. To evaluate predictors of sustained efficacy after HPS-PVP, logistic regression analysis was used. A *P* value of < 0.05 was considered statistically significant, and commercially available statistical software (SPSS 22.0, Chicago, IL) was used to analyze the data.

## Results

In total, 278 men underwent HPS-PVP during the study period. Of those, 159 (57.2%) patients with complete preoperative and postoperative follow-up clinical information were included and analyzed in this study.

The mean age of the subjects was 66.4 ± 7.1 years, and their mean body mass index was 24.1 ± 2.9 kg/m^2^. Twenty-one (13.2%) patients had diabetes mellitus, and 75 (47.2%) had hypertension. The preoperative mean PSA was 3.5 ± 4.5 ng/ml. The mean total prostate volume and transitional zone volume was 49.4 ± 32.6 ml and 29.9 ± 28.0 ml, respectively. The preoperative total IPSS was 18.9 ± 8.4, and the voiding symptom subscores and storage symptom subscores were 11.2 ± 5.5 and 7.7 ± 3.6 respectively. The QoL score was 4.0 ± 1.3. The preoperative mean Q_max_ was 10.0 ± 4.2 ml/s, and the preoperative mean PVR was 80.2 ± 79.7 ml. As shown by the preoperative urodynamic study, the mean maximal urethral closure pressure was 89.0 ± 28.0 cmH_2_O, and the maximum cystometric capacity was 408.3 ± 83.6 ml. Poor bladder compliance and involuntary detrusor contraction were present in 48 (30.2%) and 35 (22.0%) patients, respectively. The BOO index was 31.9 ± 27.3. The average operative time, energy used, and postoperative urethral catheter indwelling time were 67.4 ± 45.9 minutes, 89.6 ± 82.5 kJ and 24.5 ± 11.0 hours, respectively (Table 1).

**Table 1:** Preoperative and perioperative profiles of all patients.

Preoperative profiles	Mean ± SD or Number (Percentage)
Patients demographics	
Age (year)	66.4 ± 7.1
BMI (kg/m^2^)	24.1 ± 2.9
Comorbidities	
DM	21 (13.2%)
HTN	75 (47.2%)
PSA (ng/mL)	3.5 ± 4.5
Prostate volume (mL)	49.4 ± 32.6
Transitional zone volume (mL)	29.9 ± 28.0
Symptom Scores	
Total IPSS score	18.9 ± 8.4
Voiding Symptom subscore	11.2 ± 5.5
Storage Symptom subscore	7.7 ± 3.6
Quality of life score	4.0 ± 1.3
IIEF-5 score	8.6 ± 7.6
Uroflowmetry	
Q_max_ (mL/sec)	10.0 ± 4.2
Post void residual urine (mL)	80.2 ± 79.7
Urodynamic Study	
MUCP (cmH_2_O)	89.0 ± 28.0
First desire to void (mL)	162.8 ± 74.5
Normal desire to void (mL)	238.5 ± 96.1
Strong desire to void (mL)	340.0 ± 90.0
Maximal cystopetric capacity (mL)	408.3 ± 83.6
Pressure at Q_max_ (cmH_2_O)	45.9 ± 27.5
Poor compliance	48 (30.2%)
Involuntary detrusor contraction	35 (22.0%)
Bladder outlet obstructive index	31.9 ± 27.3
Bladder contractility index	83.8 ± 25.7
Perioperative profiles	
Operative time (min)	70.7 ± 47.6
Laser energy (kJ)	129.6 ± 99.9
Catheter duration (hr)	22.4 ± 7.3

**Fig 1:**
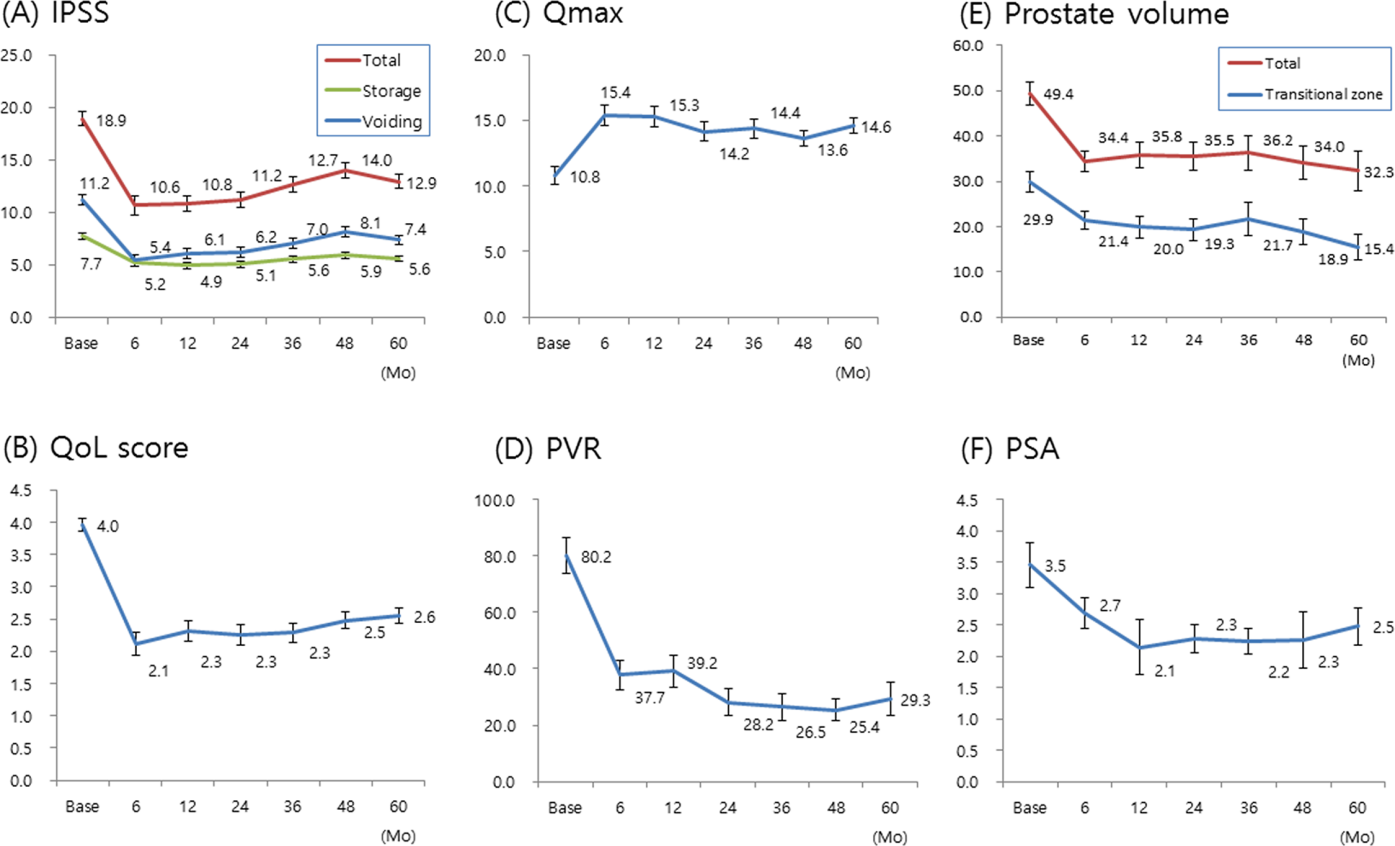
(A) Change in mean total IPSS, voiding and storage symptom subscores from baseline to 36 months postoperatively; (B) QoL scores; (C) maximal flow rates (Q_max_); (D) PVR urine volume; (E) PSA; and (F) Total prostate volume and transitional zone volume.

The IPSS/QoL scores, and Q_max_/PVR values were markedly improved from the 6-month follow-up and sustained during the course of the 5-year follow up. The reduction in the total prostate volume and transitional zone volume were also sustained after the surgery. The postoperative PSA level was significantly decreased and sustained lower than the preoperative level (Fig 1). The postoperative success rate was assessed as 101/123 (82.1%), 118/146 (80.8%) and 121/159 (76.1%) for each 1-, 3-, 5-year after surgery (Table 2).

**Table 2:** Criteria for determining the 1-, 3-, and 5-yr efficacy of individual domains (symptoms, QoL, and function) and proportion of patients for each efficacy grade.

	Postoperative Efficacy	1yr outcome	3yr outcome	5yr outcome
Symptom	Post/pre ratio of IPSS			
Excellent	≤0.25	21/117 (17.9%)	32/139 (23.0%)	26/153 (17.0%)
Good	≤0.50	23/117 (19.7%)	34/139 (24.5%)	39/153 (25.5%)
Fair	≤0.75	33/117 (28.2%)	29/139 (20.9%)	32/153 (20.9%)
Poor	>0.75	29/117 (24.8%)	44/139 (31.7%)	56/153 (36.6%)
QoL	Pre-post of QoL index			
Excellent	≥4	19/113 (16.8%)	33/142 (23.2%)	25/156 (16.0%)
Good	3	48/113 (42.5%)	50/142 (35.2%)	52/156 (33.3%)
Fair	2,1	21/113 (18.6%)	25/142 (17.6%)	26/156 (20.9%)
Poor	≤0	25/113 (22.1%)	34/142 (23.9%)	43/156 (36.6%)
Function	Post-pre of Q_max_ (mL/sec)			
Excellent	≥10	38/106 (35.8%)	18/82 (22.0%)	13/92 (14.1%)
Good	≥5	16/106 (15.1%)	6/82 (7.3%)	16/92 (17.4%)
Fair	≥2.5	32/106 (30.2%)	35/82 (42.7%)	26/92 (28.3%)
Poor	<2.5	31/106 (29.2%)	23/82 (28.0%)	37/92 (40.2%)
Success rate		101/123 (82.1%)	118/146 (80.8%)	121/159 (76.1%)

Overall efficacy is the median of efficacy grades of 3 domains.

Success definition: The overall efficacy demonstrate an improvement that was “fair or greater”

QoL, quality of life; Q_max_, maximum flow rate

Among the preoperative variables that were suspected of affecting HPS-PVP outcomes, presence of diabetes (OR 0.210, *P =* 0.017), and higher QoL scores (OR 1.554, *P =* 0.033) were independent risk parameters for the 1-yearear treatment success. Higher QoL score showed marginal clinical significance (OR 1.456, *P =* 0.054), and higher bladder outlet obstructive index were the independent predictors of the 3-year treatment success. Higher voiding symptom scores (OR 1.157 *P =* 0.022), and lower maximal cystometric capacity (OR 0.993 *P =* 0.021) from urodynamic study were the independent parameters for predicting 5-year treatment success (Table 3).

**Table 3:** Univariate and multivariate logistic regression analysis of preoperative predictors at 1-, 3- and 5-year postoperative success.

	Univariate		Multivariate	
	OR (95% CI)	P-value	OR (95% CI)	P-value
1-yr success				
Age	0.996 (0.929–1.067)	0.906		
BMI	0.973 (0.821–1.154)	0.756		
**DM (+)**	**0.184 (0.058–0.583)**	**0.004**	**0.210 (0.058–0.757)**	**0.017**
HTN (+)	0.906 (0.360–2.278)	0.833		
PSA	0.997 (0.885–1.123)	0.963		
Prostate volume	1.001 (0.987–1.015)	0.892		
Voiding Symptom subscore	1.026 (0.938–1.122)	0.579		
Storage Symptom subscore	9.993 (0.865–1.139)	0.915		
**Quality of life score**	**1.379 (0.982–1.987)**	**0.063**	**1.554 (1.036–2.332)**	**0.033**
Q_max_	0.966 (0.897–1.039)	0.353		
**Post void residual urine**	**1.007 (0.998–1.016)**	**0.124**	**1.009 (0.999–1.019)**	**0.093**
MUCP	1.002 (0.985–1.020)	0.802		
Maximal cytometric capacity	1.000 (0.994–1.006)	0.968		
Poor compliance	0.533 (0.201–1.416)	0.207		
**Involuntary detrusor contraction (+)**	**0.458 (0.167–1.258)**	**0.130**	**0.572 (0.185–1.768)**	**0.332**
Bladder outlet obstructive index	1.003 (0.983–1.022)	0.801		
Bladder contractility index	1.013 (0.990–1.036)	0.265		
3-yr success				
Age	0.987 (0.930–1.048)	0.670		
BMI	1.074 (0.928–1.244)	0.338		
DM (+)	0.808 (0.244–2.672)	0.726		
HTN (+)	0.884 (0.370–1.925)	0.686		
PSA	1.066 (0.929–1.222)	0.362		
Prostate volume	1.012 (0.995–1.029)	0.167		
Voiding Symptom subscore	1.055 (0.976–1.141)	0.176		
Storage Symptom subscore	1.038 (0.924–1.166)	0.533		
**Quality of life score**	**1.551 (1.119–2.151)**	**0.008**	**1.456 (0.993–2.133)**	**0.054**
Q_max_	0.955 (0.888–1.027)	0.213		
**Post void residual urine**	**0.996 (0.992–1.001)**	**0.128**	**0.997 (0.992–1.002)**	**0.267**
**MUCP**	**0.988 (0.973–1.003)**	**0.126**	**0.996 (0.975–1.014)**	**0.413**
**Maximal cytometric capacity**	**0.994 (0.988–0.999)**	**0.025**	**0.998 (0.992–1.005)**	**0.645**
Poor compliance	0.958 (0.389–2.360)	0.925		
Involuntary detrusor contraction (+)	0.785 (0.295–2.088)	0.627		
**Bladder outlet obstructive index**	**1.019 (0.997–1.041)**	**0.088**	**1.034 (1.004–1.064)**	**0.024**
Bladder contractility index	1.007 (0.988–1.027)	0.448		
5-yr success				
Age	0.964 (0.913–1.018)	0.188		
BMI	0.914 (0.802–1.043)	0.181		
DM (+)	1.389 (0.437–4.414)	0.577		
HTN (+)	0.750 (0.361–1.557)	0.440		
PSA	0.990 (0.915–1.072)	0.806		
Prostate volume	0.999 (0.988–1.010)	0.877		
**Voiding Symptom subscore**	**1.089 (1.015–1.168)**	**0.018**	**1.157 (1.021–1.311)**	**0.022**
**Storage Symptom subscore**	**1.082 (0.976–1.200)**	**0.134**	**0.886 (0.746–1.051)**	**0.165**
**Quality of life score**	**1.655 (1.227–2.333)**	**0.001**	**1.391 (0.909–2.128)**	**0.128**
Q_max_	0.966 (0.922–1.013)	0.152		
Post void residual urine	1.003 (0.997–1.008)	0.340		
MUCP	0.998 (0.984–1.011)	0.747		
**Maximal cytometric capacity**	**0.994 (0.989–0.999)**	**0.016**	**0.993 (0.987–0.999)**	**0.021**
Poor compliance	0.918 (0.418–2.016)	0.831		
Involuntary detrusor contraction (+)	0.729 (0.313–1.698)	0.464		
Bladder outlet obstructive index	1.009 (0.992–1.026)	0.293		
**Bladder contractility index**	**0.998 (0.976–1.006)**	**0.141**	**0.990 (0.973–1.008)**	**0.297**

Thirty-eight (23.9%) patients had immediate postoperative complications, which were managed successfully using nonsurgical methods. Due to hematuria, three (1.9%) patients underwent catheterization and irrigation, but none required a transfusion. Two (1.2%) patients complained of postoperative voiding difficulty due to bladder neck contracture or urethral stricture, and this was resolved by endoscopic reoperation. Five patients (3.1%) underwent HPS-PVP reoperation due to sustained voiding symptoms.

## Discussion

As mentioned earlier, over the past decade, laser techniques have been developed for BPH as a treatment alternative to TURP and open prostatectomy. GreenLight PVP was introduced in the late 1990s and is now commonly used for symptomatic BPH due to the availability of higher-energy lasers [8, 9]. There are very few studies of long-term outcomes after 120-W HPS-PVP compared with other endoscopic surgery modalities [7, 8].

In this study, the postoperative success rate was assessed as 82.1%, 80.8% and 76.1% for each 1-, 3-, and 5-year after surgery. Most of the postoperative parameters showed the best results in the first year after PVP, and thereafter it gradually declined. Although the presence of diabetes was important predictor for the 1-year postoperative success, preoperative BPH symptom scores and couple of urodynamic findings could help to predict 3- and 5-year postoperative good efficacy.

Since Malek et al. reported the first long-term follow-up results of 80-W laser PVP, several reports of long-term outcomes have been published [21–24]. In a prospective 5-year follow-up study by Guo et al. of 80-W laser PVP, the reoperation rate due to urethral stricture (13%), bladder neck contracture (3%), and persistent or recurrent adenoma (18%) were much higher than other long-term results [23]. When we compared our study population with that in the study by Guo et al., although the baseline characteristics of the subjects in both studies were similar, the perioperative data, including operation times, laser energy used, and catheter indwelling times, were quite different. The operative times were longer in their study, and the laser energy used was greater. In addition, urethral catheters were required for a longer time during the perioperative period. They attributed these unsatisfactory outcomes to the inefficient tissue vaporization capacity of the former 80-W laser technique [23].

Yamada et al. reported long-term surgical outcomes and safety in a 10-year follow-up study of PVP [24]. Including repeated PVP and endoscopic internal urethrotomy, they reported a reoperation rate of 4.7%, which was comparable to the rate (4.3%). in the present study. They used both 80-W and 120-W laser PVP and concluded that the applied energy (≥ 250 kJ), preoperative IPSS (≥ 20), operation time (≥ 75 minutes), and prostate volume (≥ 80 ml) were independent significant risk factors for re-treatment after PVP [24].

The excellent hemostatic capability of GreenLight PVP is well known, even in patients with anticoagulation [7, 25]. In this study, none of the patients required transfusions. The postoperative complications in the present study were similar to those reported in other long-term series and included dysuria and mild hematuria [7, 25]. In addition, most of the cases could be managed conservatively, and the rate of urethral catheter re-indwelling and transfusion were very low (0–2%).

Hueber et al. reported that the difference in the prostate volume did not influence the efficacy and safety of PVP [26]. This was also the case in the present study, in which there was no association between postoperative voiding parameters and improvements in the prostate volume. The prostate volume showed a positive correlation only with the total operative time and applied energy. Postoperative complications, such as urethral stricture or bladder neck contracture, and the re-operation rate were not associated with the prostate volume.

The present study has some limitations due to its retrospective study design. Although most patients adhered to the prescheduled regular follow-up plan, some did not. Thus, data are missing on some patients at certain follow-up points. The drop-rate at the 5-year follow up was 42.8% (128/289). Thus, there could be a risk of selection bias and under- or over-estimation. In other words, when the patients who were not satisfied at the postoperative outcome, some patients might tend to be continuously tracked and observed or other patients might go to another hospitals and the follow-up would be interrupted. Nevertheless, to our knowledge, this is the first 5-year long-term study of a large number of patients with BPH after 120-W HPS-PVP. And this study results also provided the postoperative follow up rate after BPH surgery in real practice.

## Conclusion

HPS-PVP is an effective and durable procedure for the treatment of BPH. The postoperative success rate was assessed as 82.1%, 80.8% and 76.1% for each 1-, 3-, 5-year after surgery. Presence of DM, voiding symptom subscore, QoL, Maximal cystometric capacity, and BOO index were valuable preoperative parameters for predicting postoperative success.

## Supporting information

S1 File Patient dataset. (XLS)
